# Evaluation of the Impact of Esterases and Lipases from the Circulatory System against Substrates of Different Lipophilicity

**DOI:** 10.3390/ijms23031262

**Published:** 2022-01-23

**Authors:** Leslie Lam, Marc A. Ilies

**Affiliations:** 1 College of Science and Technology, Temple University, 1803 N. Broad Street, Philadelphia, PA 19122, USA; leslie.lam@temple.edu; 2 Department of Pharmaceutical Sciences and Moulder Center for Drug Discovery Research, Temple University School of Pharmacy, 3307 N Broad Street, Philadelphia, PA 19140, USA; 3 Lewis Katz School of Medicine, Alzheimer’s Center (ACT), Temple University, Philadelphia, PA 19140, USA

**Keywords:** esterase, lipase, ester hydrolysis, kinetics, assay, substrate lipophilicity, structure-property relationships

## Abstract

Esterases and lipases can process amphiphilic esters used as drugs and prodrugs and impact their pharmacokinetics and biodistribution. These hydrolases can also process ester components of drug delivery systems (DDSs), thus triggering DDSs destabilization with premature cargo release. In this study we tested and optimized assays that allowed us to quantify and compare individual esterase contributions to the degradation of substrates of increased lipophilicity and to establish limitations in terms of substrates that can be processed by a specific esterase/lipase. We have studied the impact of carbonic anhydrase; phospholipases A1, A2, C and D; lipoprotein lipase; and standard lipase on the hydrolysis of 4-nitrophenyl acetate, 4-nitrophenyl palmitate, DGGR and POPC liposomes, drawing structure–property relationships. We found that the enzymatic activity of these proteins was highly dependent on the lipophilicity of the substrate used to assess them, as expected. The activity observed for classical esterases was diminished when lipophilicity of the substrate increased, while activity observed for lipases generally increased, following the interfacial activation model, and was highly dependent on the type of lipase and its structure. The assays developed allowed us to determine the most sensitive methods for quantifying enzymatic activity against substrates of particular types and lipophilicity.

## 1. Introduction

Humans evolved to be able to consume a large variety of foods, which is essential for ensuring a good balance of essential amino acids, vitamins and minerals needed for a good health. These nutrients are efficiently absorbed in the gastrointestinal tract and transported through the circulatory system to tissues where they are needed and consumed. Together with nutrients, many natural amphiphilic compounds present in food are absorbed. Since many of them might be harmful, our circulatory system contains enzymes able to break down these xenobiotics to harmless components. One of these enzymatic systems is represented by esterases and lipases, which hydrolyze esters into corresponding acids and alcohols, which are much more polar and can be either used in the process metabolism or excreted, as needed [1,2]. Human serum albumin, which is an amphiphilic 66 KDa protein present in blood at a very high concentrations (as high as 50 mg/mL), acts as a sponge for any amphiphilic compound, thus permitting blood esterases and lipases enough time to hydrolyze them and detoxify the circulatory system [3]. Albumin is also reported to possess weak esterase activity; thus, it is able to directly hydrolyze amphiphilic esters bound to it [4]. Amphiphilic compounds that can cross membranes can also be hydrolyzed by carbonic anhydrase, a hydrolase with significant esterase activity present in large amounts of erythrocytes and also present on the surface of the lung and brain capillaries [5,6,7]. The same amphiphilic compounds can also be processed by various lipases associated with or secreted in the vicinity of biological membranes [8]. Obviously, these esterases and lipases are relevant for the pharmacokinetic and biodistribution of amphiphilic esters used as drugs and prodrugs [9]. Additionally, esterases and lipases of the circulatory system are able to process ester components of drug delivery systems (DDSs), thus triggering DDSs destabilization with premature cargo release [10,11]. Therefore, in order to develop drugs, prodrugs and DDSs with well-defined stability in the circulatory system, it is essential to understand the factors affecting the interaction of these entities with different esterases and lipases present in this environment. The first step towards this general goal is the development of assays able to quantify and compare the action of different circulatory system esterases and lipases on substrates with different lipophilicities, as this is important for determining protein catalysts that can destabilize them while in circulation. Therefore, we proceeded to quantify the action of key esterases and lipases found in the circulatory system on substrates of increased lipophilicities. The goal of this study was to test and optimize assays that permit quantifying individual esterase contributions, directly and indirectly, to the degradation of substrates of increased lipophilicity and to establish their limitations in terms of substrates that can be processed by a specific esterase/lipase.

## 2. Results and Discussion

### 2.1. General Considerations Related with Assay Development

The development of assays able to quantify the action of different circulatory system esterases and lipases is of crucial importance for determining protein catalysts that can destabilize drugs, prodrugs and DDSs while in circulation. When developing a functional assay, one should consider enzymes, substrates, cofactors and buffers and the detection methods available.

For enzymes, the specific type, activity and stability are important factors to consider while developing an assay. Purity of the enzyme is another factor to consider, since impure enzyme preparations may result in inefficient binding relative to targeted substrates. We have selected only purified enzymes from commercial sources in order to facilitate the reproducibility of developed assays.

Similarly to enzymes, the specific type of substrate chosen is important for the correct assessment of enzymatic activity. Substrate concentration affects enzymatic activity and correlates to the signal intensity of the assay. Solubility limitations affect the ability of the substrate to dissolve in an assay buffer. We have selected established and commercially available substrates for esterases and lipases for our study, such as 4-nitrophenyl acetate (4NPA), 4-nitrophenyl palmitate (4NPP), 1,2-di-O-lauryl-rac-glycero-3-glutaric acid 6′-methylresorufin ester (DGGR) and 1-palmitoyl-2-oleoyl-glycero-3-phosphocholine (POPC) of increased lipophilicity, in order to assure the reproducibility of these assays in different labs.

Most enzymes need cofactors to function properly, and the binding of specific cofactors can dramatically enhance enzymatic activities. Cofactors can be left unchanged or can cycle through different states in a reaction and are not consumed in the overall reaction. They consist of metal ions bound to active sites, coordinated with the enzyme, or organic compounds used for hydride transfer. Cofactors are normally added to assays in order to maximize enzymatic activity. Their concentrations are important as cofactors may obstruct the detection of products produced in enzymatic reactions. Calcium ion is an important cofactor for many lipases; therefore, it was added to the assay in concentrations that are physiologically relevant.

Buffers also have a significant impact on assay conditions since the type of buffer used can affect specific enzyme activities, which can translate in non-optimal assay conditions and affect the reproducibility of the assay. Many in vitro assays were performed at physiological pH to emulate intracellular conditions of native enzymes. Enzymes are sensitive to pH and will denature at high or low pH levels. Some buffers commonly used in enzymatic assays include tris(hydroxymethyl)aminomethane (Tris), 4-(2-hydroxyethyl)-1-piperazineethanesulfonic acid (HEPES), 3-(N-morpholino)propanesulfonic acid (MOPS), sodium and potassium phosphate; therefore, they were preferred in the assays developed [12].

Lastly, the type of detection method used to assess enzyme activity depends on throughput sensitivity, cost, assay robustness, nature of reaction that is being studied and on the substrates and products that require quantification. All detection methods have their advantages and disadvantages. Continuous reactions require directly monitoring reactions and typically use a spectrophotometer or fluorometer to collect data from multiple time points, which was the case in our developed assays. Moreover, this process helps in the detection of substrates or products that absorb or emit light. Many systems use absorbance, fluorescence or changes in light scattering for the detection of enzymatic activity. The procedures used included observing the entire reaction over a specific time period after mixing the enzyme and substrate. This minimizes the error and need for transfers of reaction components in or out of the system [12].

### 2.2. Esterase and Lipase Activity Overview and Selection of Representatives

Esterases are important enzymes that catalyze the hydrolysis of ester bonds of water-soluble substrates into corresponding alcohol and acid precursors. An important esterase present in the circulatory system is carbonic anhydrase (CA), a zinc metalloenzyme with 15 isozymes [5]. In its main catalyzed reaction, CAs reversibly hydrate carbon dioxide into bicarbonate ion and a proton. This reaction is important for physiological and pathological processes such as respiration and transport of carbon dioxide and bicarbonate between tissues and lungs; pH and carbon dioxide hemostasis; electrolyte secretion in tissues and organs; and biosynthetic reactions. However, many carbonic anhydrase isozymes, such as CA I and CA II isozymes present in large amounts in red blood cells, or CA IV and CA XII present in the lung and brain endothelial capillaries possess esterase capabilities, and they are able to process a diverse range of esters [6,13,14]. Therefore, CA was considered representative for processing blood esterases and low molecular weight esters, and CA II was included in our set of enzymes to be investigated.

Lipases are a category of esterases that catalyze the hydrolysis of esters from insoluble water or heavily aggregated substrates such as phospholipids or triglycerides composed of long chain fatty acids through interfacial activation [2]. For example, phospholipases 1 and 2 (PLA1 and PLA2) hydrolyze 1,2-diacyl-sn-glycero-3-phospholipids at either the sn-1 position (PLA1) or at the sn-2 position (PLA2). The related congener phospholipase C (PLC) hydrolyzes the same 1,2-diacyl-sn-glycero-3-phospholipids glycerophosphate bond, while phospholipase D (PLD) cleaves the terminal phosphodiester bond of the phospholipids, yielding phosphatidic acid [15]. Lipases, such as PLA1, PLA2, PLC and PLD outlined above, evolved to process natural amphiphiles, but they can also process their synthetic congeners; therefore, they were also considered in our study. Moreover, in the human body, natural lipases participate in various processes such as digestion, absorption and transportation of dietary triglycerides, which includes the dynamic processing of low-density and high-density lipoprotein, thus playing an important role in cholesterol trafficking from the liver to the tissues and vice versa [16,17]. Pancreatic lipase and related lipases efficiently hydrolyze hydrophobic fatty acid esters of glycerol (fats), breaking them down into components that are easily absorbed, and this prompted us to select a prototypical lipase such as lipase from P. cepacia (LPC) in our study. In the same context, one can emphasize that lipoprotein lipases (LPLs) hydrolyze triglycerides present in the vascular system that are transported by chylomicrons and very low-density lipoproteins (natural delivery systems for fats and cholesterol). LPLs are secreted as glycoproteins, which are anchored to the surface of endothelial cells via heparin sulfate proteoglycans. This allows LPLs to be in contact with lipoproteins that are moving in the vascular system. Once in contact with them, they hydrolyze lipoproteins into fatty acids, and the fatty acids are absorbed by the surrounding tissues [8,17]. Therefore, an LPL representative was included in our study.

### 2.3. Hydrolysis of 4-Nitrophenyl Acetate Mediated by Esterases and Lipases

**Figure 1:**
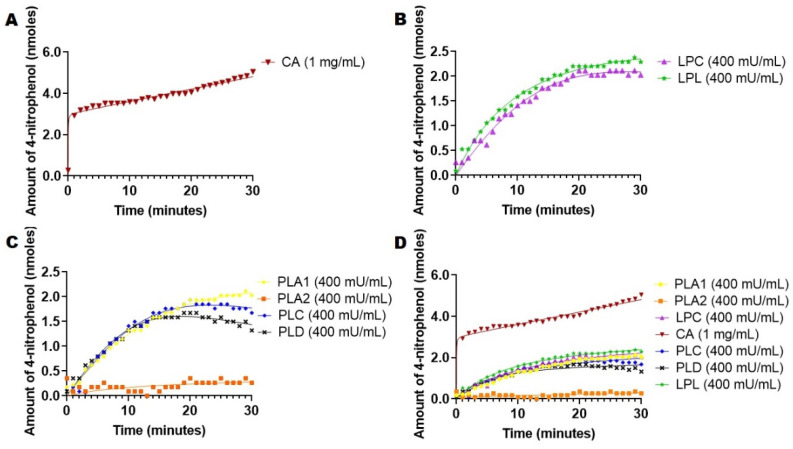
Hydrolysis of 4-nitrophenol acetate catalyzed by different esterases present in the circulatory system at 400 mU/mL (except CA (1 mg/mL)). Enzymatic kinetics were recorded via absorbance at 400 nm. The uncatalyzed hydrolysis of 4-nitrophenyl acetate was deducted from the rate of catalyzed reaction in each case. The amount of 4-nitrophenol released was determined using a calibration curve. (**A)**. Hydrolysis with CA. (**B**). Hydrolysis with lipases (LPC and LPL). (**C**). Hydrolysis with Phospholipases (A1, A2, C and D). (**D**). Hydrolysis with all esterases and lipases combined.

We have considered 4-nitrophenyl acetate because it is an amphiphilic substrate commonly used for many esterases [13,18,19]. Exploiting the amphiphilicity of 4-nitrophenyl acetate as a substrate under the sensitivity of this assay (due to high extinction coefficient of 4-nitrophenolate product), we applied this assay to several esterases that are present in the circulatory system: CA, PLA1, PLA2, LPC, PLC, PLD and LPL. Esterase activities for different enzymes (total assay 400 mU/mL) were used in all cases except CA (1 mg/mL) in order to permit the comparison of the intrinsic activity of the enzyme relative to amphiphilic substrate. The rates of hydrolysis of 4-nitrophenol acetate were recorded from the above-mentioned enzymes, and the hydrolysis reaction was monitored for up to 30 min and every minute at 37 °C (Figure 1).

For the case of PLA2, the assay was supplemented with Ca^2+^ since the activity of the enzyme is calcium dependent [20,21], and the assay was also supplemented with BSA to bind the fatty acid produced during the hydrolysis process [21]. The uncatalyzed hydrolysis at the same working concentration was recorded at 37 °C every minute for 30 min, and it was deducted from the rate of catalyzed reactions. A calibration curve of 4-nitrophenol was performed to determine the amount of 4-nitrophenol released from the enzymes. The absorbances for the catalytic activity were converted to the amount of nanomoles product per liter released, using calibration curves (Supplementary Materials, Figures S1 and S2). The results are summarized in Figure 1.

Data from Figure 1a show that CA released a significant amount of 4-nitrophenol of 5 nmols in 30 min at working concentrations with a non-hyperbolic curve. Phospholipases displayed lower activities and released less than 3 nmols of 4-nitrophenol at the same time (Figure 1B). Lipases LPL and LPC demonstrated the same activity as active phospholipases, releasing about 2 nmol 4-nitrophenol within 30 min (Figure 1C). This relative processivity of the enzymes is revealed in the combined graph (Figure 1D).

Previous studies showed that 4-nitrophenyl acetate was commonly used as the substrate for studying CA activities due to the similarity between CO_2_ hydrolysis reactions, which could explain how CA hydrolyzed and released the highest amount of 4-nitrophenol [13,19]. A study on esterase, phosphatase and sulfatase activities on CA isozymes showed that CA isozymes prefer 4-nitrophenyl acetate as the substrate compared to 4-nitrophenyl phosphate or 4-nitrophenyl sulfate due to the strong nucleophilic attack of Zn^2+^ and OH without electrostatic repulsion [13]. Thorslund et al. performed similar experiments and tested various nitrophenyl derivatives with bovine erythrocytes CA and obtained similar results compared to previously published results. They found that the hydrolysis rate decreased substantially (5000 times) for p-nitrophenyl trimethylacetate as compared with the standard 4-nitrophenyl acetate [22]. For CA, the shape of the curve is not the typical hyperbolic curve obtained for most enzymes. It shows a rapid burst of 3 nmols of 4-nitrophenol released during the first minute followed by a steady and slow release. Gutfreund and Sturtevant performed an experiment to test the hydrolysis of 4-nitrophenyl acetate with chymotrypsin. They discovered that, initially, there was a rapid liberation of one mole of p-nitrophenol per mol of chymotrypsin followed by slow hydrolysis [23]. Nelson also confirms that this is a typical curve for 4-nitrophenol. He explains that the release of 4-nitrophenol reaction releases a rapid burst of 4-nitrophenol that is nearly stoichiometric with the amount of enzyme that is present, which reflects the fast acylation phase of the reaction [24]. Afterward, the subsequent rate is slower because enzyme turnovers were limited by the rate of the deacylation phase [24]. The study from Burdette et al. explained the importance of interfacial reaction dynamics on LPL-mediated hydrolysis of 4-nitrophenyl esters. The authors concluded that LPL has higher specificity for intermediate chain lengths, which can explain why LPL has lower activity with 4-nitrophenyl acetate than compared with 4-nitrophenol palmitate (see below) [25]. LPL, LPC, PLA1, PLC and PLD had similar activities, displaying approximately the same amount of 4-nitrophenol released, whereas PLA2 was the only enzyme that had no activity in the presence of 4-nitrophenyl acetate in our assay conditions, a fact that was attributed in the literature to its specific catalysis mechanism, which depended significantly on the lipophilicity of the substrate [26].

### 2.4. Hydrolysis of 4-Nitrophenyl Palmitate Mediated by Esterases and Lipases

4-Nitrophenyl palmitate was chosen as the substrate because it is much more lipophilic than 4-nitrophenyl acetate. A literature search provided insights on how the assay would be conducted by preincubating the enzyme in a buffer and inducing the enzymatic reaction by the addition of substrates [27]. A typical concentration of 10 mM 4-nitrophenyl palmitate stock was used for the assay [28]. The addition of a small amount of Triton X-100 additive is a method for eliminating turbidity in an assay, since many researchers reported problems with spectrophotometric measurements when using this substrate [27]. Since 4-nitrophenol palmitate is not soluble in water, we decided to follow these reports and add Triton X-100 detergent at 1% final concentration in assay. Triton X-100 efficiently dissolved 4-nitrophenyl palmitate and helped stabilize micellar substrates in the absence and presence of enzymes.

**Figure 2:**
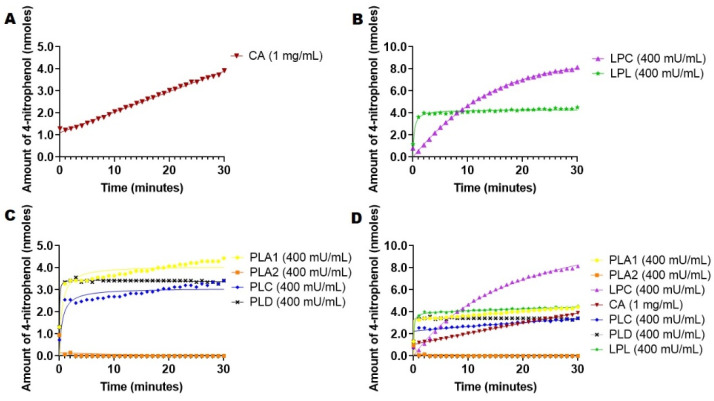
Hydrolysis of 4-nitrophenol palmitate catalyzed by different esterases present in the circulatory system at 400 mU/mL except CA (1 mg/mL). Enzyme kinetics were recorded via absorbance at 400 nm. The uncatalyzed hydrolysis of 4-nitrophenyl palmitate was deducted from the rate of catalyzed reaction observed. The amount of 4-nitrophenol released was determined against a calibration curve (Supplementary Materials, Figures S3 and S4). (**A**) Hydrolysis with CA. (**B**) Hydrolysis with lipases (LPC and LPL). (**C**) Hydrolysis with Phospholipases (A1, A2, C and D). (**D**) Hydrolysis with all esterases and lipases combined.

We applied the Triton X-100-based 4-nitrophenyl palmitate assay to the esterases mentioned above in similar assay conditions used for 4-nitrophenyl acetate congener (400 mU/mL), except for CA (1 mg/mL), in order to compare the intrinsic activity of the esterases with the lipophilic substrate. Assay conditions were similar to the ones used in the case of 4-nitrophenyl acetate, and the hydrolysis of catalyzed reaction was recorded at 37 °C for 30 min, reading the absorbance every minute. For PLA2, the assay was supplemented with CaCl_2_ and BSA that bind the palmitate released in the reaction [20,21,29]. Uncatalyzed hydrolysis at same working concentration was recorded at 37 °C for 30 min, measuring the absorbance every minute and deducting it from the absorbance values obtained for the catalyzed reactions (Figure 2).

Data from Figure 2A showed that hydrolysis of 4-nitrophenyl palmitate in Triton X-100 micelles in the presence of carbonic anhydrase progressed at a slower rate than in the case of 4-nitrophenyl acetate (Figure 1A). In contrast, phospholipases displayed slightly higher activities (except for PLA2, Figure 2B) than compared with 4-nitrophenyl acetate hydrolysis (Figure 1B). It was also revealed that LPC had the highest catalytic activity in this assay with a distinct hyperbolic curve compared to the other esterases and LPL, releasing 8 nmols 4-nitrophenol in 30 min (Figure 2C,D). Ganasen et al. studied the hydrolysis of 4-nitrophenyl palmitate with LPC on an immobilized support, at various substrate concentrations, and determined the hyperbolic curves of activity for all substrate concentrations, which are similar to our findings (Figure 2B) [30].

**Figure 3:**
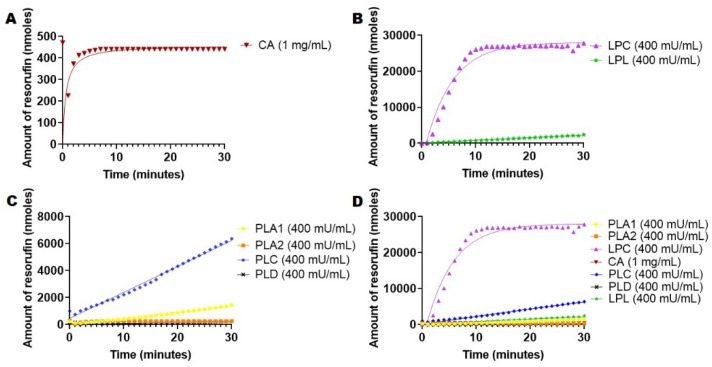
Hydrolysis of DGGR of lipases and esterase (80 U/mL) in 20 mM Tris-HCl, 150 mM NaCl and 0.05% Triton X-100 and 800 µg DGGR. The amount of resorufin produced was determined via absorbance at 572 nm. (**A**) Hydrolysis with CA. (**B**) Hydrolysis with lipases (LPC and LPL). (**C**) Hydrolysis with Phospholipases (A1, A2, C and D). (**D**) All hydrolysis kinetics (esterases and lipases) combined.

A comparison of LPC-mediated hydrolysis of different 4-nitrophenyl derivatives showed the LPC has a higher specific activity for 4-nitrophenyl palmitate, 4-nitrophenyl caprylate and 4-nitrophenyl butyrate [31]. Bornscheuer et al. crystalized LPC and determined that LPC has high preference of triglycerides and saturated fatty acid esters such as stearyl and palmityl ester [32]. This is consistent with our data that show that LPC released higher amounts of 4-nitrophenol when 4-nitrophenyl palmitate was used as the substrate. (Figure 1B and Figure 2B). LPC also showed a hyperbolic curve for 4-nitrophenyl acetate hydrolysis (Figure 1). Kim et al. studied the crystal structure of LPC and found that LPC has an open conformation at the lipid–water interface, which shows its higher processivity without inhibition (Figure 3B) [28]. This proves that LPC prefers lipophilic substrates than hydrophilic ones (Figure 1 and Figure 2). LPL and PLA1 had a similar catalytic activity of 4-5 nmoles 4-nitrophenol released in 30 min. However, CA, PLC and PLD all released 3-4 nmoles in 30 min. One can observe that PLA1 and LPL catalytic activity doubled when 4-nitrophenylpalmitate was used as the substrate, as compared to the hydrolysis of 4-nitrophenyl acetate.

While esterases efficiently catalyze the hydrolysis of water-soluble substrates and follow the Michaelis–Menten mode, lipases display interfacial activation [33]. Sandra et al. demonstrated the difference between esterase and lipase based on their ability to be activated by interfaces. They discovered that the unique characteristic of lipases is interfacial catalysis. Their experiments showed that pancreatic lipases showed activity when triacetin, a water-soluble triglyceride, is in a monomeric state. When the substrate concentration reached the solubility limit, hydrolysis increases rapidly with the same substrate as micelles or was present as emulsion drops. This activity was shown to be independent of molar concentrations but dependent on the concentration of the substrate at the interface [34,35].

The structure of the enzyme is critical for achieving interfacial activation. Both PLA1 and LPL have a beta-5 loop in the catalytic domain, which can account for the similar hydrolytic activities of these enzymes [36,37]. Shirai et al. discovered that LPL hydrolyzes short-chain acyls (tributyrin, p-nitrophenyl acetate and p-nitrophenyl butyrate) [36]. Our results confirmed that the enzymes prefer lipophilic substrates in addition to the Triton detergent. Dippe et al. explored substrate recognition of PLA1 and found that the binding site strongly favors hydrophobic medium chain acyl substrates, while short chain ester 4-nitrophenyl acetate was not favored by PLA1s [38]. Another previous study showed that LPL has a phenylalanine (Phe-119) located near the binding pocket, which interacts weakly with substrates possessing less than eight carbon chains. This could explain why LPL prefers longer or intermediate chain hydrophobic substrates, as mentioned previously [25,31]. Moreover, Lehner and Verger indicated that medium chain triacylglycerols were more effectively hydrolyzed by enzymes in the presence of a detergent, emphasizing the importance of the physical state of the substrate. In the absence of a detergent, hydrolase activity decreased very sharply, which could be due to the impaired desorption of the reaction products [39]. PLD and PLC also displayed 2X activity against 4-nitrophenyl palmitate compared to 4-nitrophenyl acetate. It appears that the addition of Triton X-100 allowed improved catalytic activity. Thus, Okawa et al. studied various conditions for PLD isolated from Streptomyces species activity and concluded that PLD hydrolyzes long chain lipids (phosphatidylethanolamine, phosphatidylcholine, phosphatidylserine, sphingomyelin, cardiolipin and lysophosphatidic choline), with phosphatidylethanolamine being the best substrate when Triton X was added at low concentrations (0.1% and 0.2%) [40]. Cha et al. explored the hydrolysis of p-nitrophenyl phosphoryl derivatives by PLD and determined the kinetic parameters for different substrates of various chain lengths or polar head groups. They concluded that Vmax for long chain alky groups decreased, and it was highest for 4-nitrophenyl phosphoryl acetate, which contrasts our data and shows that charged derivatives and chain length affect hydrolysis [41]. We concluded that lipophilic substrates positively affect the activity of PLC and PLD (Figure 1 and Figure 2). Similarly to the 4-nitrophenyl acetate case, PLA2 also displayed no activity against 4-nitrophenol palmitate (Figure 1 and Figure 2).

### 2.5. Hydrolysis of 1,2-Di-O-Lauryl-rac-glycero-3-glutaric acid 6′-methylresorufin ester (DGGR) Mediated by Esterases and Lipases

We developed our DGGR assay based on the chromogenic substrate purchased from Sigma Aldrich. DGGR is a lipid-conjugated substrate, which is used as a specific substrate for lipases. A literature search was conducted to establish optimum assay conditions and procedures, which were as follows: 20 mM Tris-HCl buffer, 150 mM NaCl and 0.05% Triton X-100 [39] using DGGR from a substrate stock (2 mg/mL in dioxane). The hydrolysis of DGGR was followed via absorption at 572 nm [42,43]. Our assay was similar to other published assays involving DGGR [44,45]. We must mention that this assay can be conducted via fluorescence (for example, 529, em. 600, cutoff 590 nm) and that one can use BSA with assay conditions. We exploited the lipophilicity of DGGR as a substrate against the enzymes mentioned above to determine the intrinsic activity towards this particular lipophilic substrate. The initial rates of hydrolysis of DGGR were induced by the addition of the mentioned enzymes, recording the hydrolysis reaction for up to 30 min every minute at 37 °C via absorbance. In the case of PLA2, the assay was supplemented with Ca^2+^ since the activity of enzyme is calcium dependent [20,21,29] and with BSA to trap the fatty acid produced during the hydrolysis process [21]. Uncatalyzed hydrolysis at the same working concentration was recorded at 37 °C for 30 min every minute, and it was deducted from the rate of catalyzed reactions. Data are presented in Figure 3.

Data from Figure 3 showed that LPC released the highest amount of resorufin compared to the rest of the enzymes, peaking at about 25,000 nmols product in 30 min. (Figure 3A,D). Data from Figure 3A revealed that CA had the lowest catalytic activity, which was limited to about 400 nmols released in 30 min. PLA1 and LPL both appeared to have similar catalytic activities of about 1500 nmols and 2300 nmols produced in 30 min, respectively. PLC hydrolyzed DGGR at a rate of about 2000 nmols in 30 min. Overall, lipases and esterases were able to process and hydrolyze DGGR at a higher rate than 4-nitrophenyl acetate and 4-nitrophenyl palmitate, thus revealing the impact of substrate structure and lipophilicity on esterase and lipase activities.

### 2.6. PLA2-Specific Hydrolysis of Phospholipids

PLA2 constituted a special case, since 4-nitrophenyl derivatives assays were not usable and the DGGR assay was not sensitive enough. Therefore, we decided to pursue a more sensitive assay. A literature survey revealed that PLA2 requires cofactors such as Ca^2+^ to be active and that it is inhibited by fatty acids [20,46]. We have selected a final concentration of 1 mM CaCl_2_ and 1 mg/mL BSA in our assay to bind the fatty acid produced [21,29,46,47]. Garret et al. and Lafferty et al. also used 0.2% BSA in the DGGR assay for the same reason [44,45]. Another survey of the literature available revealed two main methods that assess PLA2 activity based on quantitation of acid liberated during hydrolysis of phospholipids. Method 1 is essentially a pH stat method that employs an autotitrator [15,48,49,50,51], while Method 2 involves bromothymol blue as a pH indicator [52,53]. We have selected bromothymol blue (BTB) as the indicator dye due to the fact that its color changes at around physiological pH (pH 6.0–7.6) [53,54]. The final assays conditions were similar to the conditions used previously, namely Buffer 1, 1 mM CaCl_2_ and 1 mg/mL BSA, in order to allow easy translation from one assay to another.

**Figure 4:**
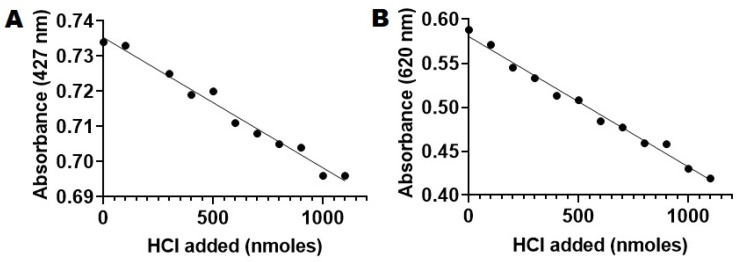
(**A**). Absorbance of BTB (0.05 mM working concentration) as a function of the amount of HCl added at 427 nm. (**B**). Absorbance of BTB (0.05 mM working concentration) as a function of the amount of HCl added at 620 nm.

We first examined the solubility of BTB in the working buffer by using two calibration curves at two absorbance maximums of 427 nm and 620 nm recorded in the UV spectrum in buffer 1 (pH 7.4 in HBS 1X, Supplementary Materials, Figure S5) [53,55]. After analysis of the calibration curves, we confirmed a linear response up to 0.1 mM at 427 nm and up to 0.07 mM at 620 nm (Supplementary Materials, Figures S6 and S7). As a result, we decided to use a working concentration of 0.05 mM of BTB, at which the dye was perfectly soluble in the working buffer and would not interfere with the other components of the assay. By using this working concentration, we calibrated the dye response to the acid by recording absorbance at two wavelengths (427 nm and 620 nm) of 0.05 mM BTB in buffer:ethanol while adding fixed amounts of 10 mM HCl (Figure 4).

**Figure 5:**
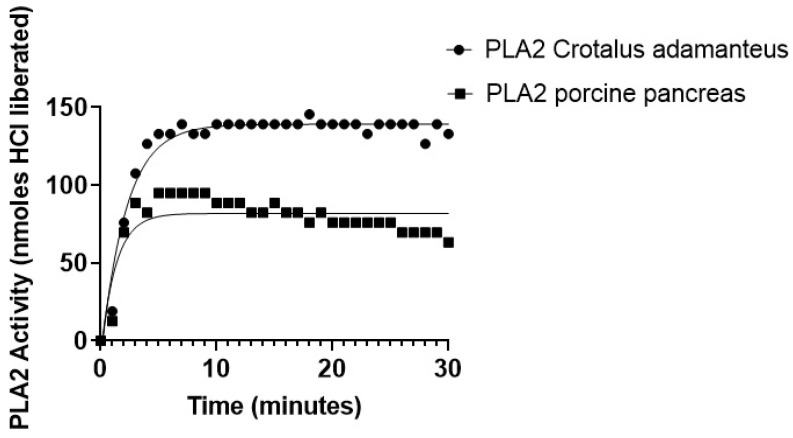
Assay of PLA2 from Crotalus adamanteus and PLA2 from porcine pancreas activity with 0.1 mM POPC liposomes, 1 mM CaCl_2_, 1 mg/mL BSA and 0.05 mM BTB. Absorbance was recorded at 620 nm and converted to nmols released.

From Figure 4A,B, one can observe that the response of BTB dye was linear in the range of 0–100 nmols of added acid at both 427 nm and 620 nm (R2 values of 0.9808 and 0.9877). This allowed us to assess PLA2 activity by using a BTB-based assay in which PLA2 was hydrolyzing POPC liposomes. The absorbance obtained from the assay was converted to moles of acid by using the calibration curves previously used (Figure 5).

The results presented in Figure 5 revealed that the addition of PLA2 from Crotalus adamanteus released about 140 BTB nmols HCl after 5 min incubation time, while PLA2 from porcine pancreas peaked at about 95 nmols of HCl was released after 5 min with a deviation from the hyperbolic curve. No additional products were formed for the rest of the study (up to 30 min incubation time, See also Supplementary Materials, Figure S8). The results can be explained by considering that Cunningham et al. studied the product inhibition of venom group IA sPLA2 in in vitro conditions with plasma and ex vivo conditions with rats, and they concluded that lysophosphatidylcholines act as uncompetitive inhibitors on sPLA2s and, at high substrate levels, caused deviations from normal hyperbolic curves [56]. They also concluded that, according to the scooting theory, sPLA2 cleaves the ester and releases lysoPC [56]. At high concentrations of free or desorbed lysoPC, it causes inhibition of enzymatic activity [56], which can help explain the curves of Figure 5.

## 3. Materials and Methods

### 3.1. Materials

Phospholipase A1 (PLA1) from Thermomyces lanuginosus (Sigma-Aldrich L3295); phospholipase A2 (PLA2) from porcine pancreases (Sigma-Aldrich P6534) and from Crotalus adamanteus (Worthington Lakewood, NJ, USA); lipase from Pseudomonas Cepacia (LPC) (Sigma-Aldrich 62309); carbonic anhydrase (CA) from bovine erythrocytes (Sigma-Aldrich C2624); lipoprotein lipase (LPL) from Burkholderia sp. (Sigma-Aldrich L9656); phospholipase C (PLC) from Clostridium perfringens (C. welchii) (Sigma-Aldrich P7633); phospholipase D (PLD) from Streptomyces chromofuscus (Sigma Aldrich P0065); and 1,2-Di-O-lauryl-rac-glycero-3-(glutaric acid 6-methylresorufin ester) (DGGR) (Sigma Aldrich 30058) were purchased from Sigma-Aldrich (St. Louis, MO, USA) and were used as received. 4-Nitrophenyl acetate was from Acros Organics (Waltham, MA, USA), and 4-nitrophenyl palmitate was purchased from Alfa Aesar (Tewksbury, MA, USA). TritonX-100 and sodium phosphate dibasic anhydrous were obtained from Fisher Scientific (Waltham, MA, USA). Tris (M151G) and HEPES free acid (0511) were obtained from AMRESCO (Allentown, PA, USA). Sodium chloride (MK7581) was from Macron Fine Chemicals (Waltham, MA, USA). 1.2-Dimethoxyethane (DX1531), chloroform (CX1055) and methanol (MX0486) were purchased from EMD Millipore (Burlington, MA, USA), and p-dioxane (9231) was from Avantor (Allentown, PA, USA). Fatty acid-free Albumin bovine serum Fraction V was purchased from Calbiochem (San Diego, CA, USA), and CaCl_2_ was purchased from Aldrich Chemical Company Inc. (Milwaukee, WI, USA). Bromothymol blue (BTB) was purchased from Tokyo Chemical Industries (Portland, OR, USA). 1-Palmitoyl-2-oleoyl-glycero-3-phosphocholine (POPC, 850457) was purchased from Avanti Polar Lipids (Alabaster, AL, USA).

Buffer 1 was HBS pH 7.4, 140 mM sodium chloride, 1.5 mM sodium phosphate dibasic anhydrous and 50 HEPES free acid in DI water (HBS 1X).

Buffer 2 was 150 mM NaCl and 20 mM Tris adjusted with 0.1 mM HCl to pH 8.0, followed by the addition of 0.5% *v*/*v* TritonX-100. Both buffers were stored at 4 °C.

Stock solutions of PLA1 (132 U/mL), PLA2 (80 U/mL), LPC (80 U/mL), LPF (80 U/mL), CA (1 mg/mL), PLC (80 U/mL), PLD (80 U/mL) and LPL (80 U/mL) were made in HBS 1X by dissolving the received powder in HBS 1X overnight, followed by filtration through 0.2 µm low binding filters. Enzyme stock solutions were kept at 4 °C.

Substrate stocks were as follows: 4-nitrophenyl acetate was 10 mM in dimethoxyethane (DME); 4-nitrophenyl palmitate was 10 mM in HBS 1X with 10% Triton X-100 sonicated for 30 min and filtered through 0.2 µm filters; and 1,2-Di-O-lauryl-rac-glycero-3-(glutaric acid 6-methylresorufin ester) (DGGR) was made 2 mg/mL in p-dioxane. All substrate stocks were stored at 4 °C prior to use.

For the PLA2 assay, stock solutions of CaCl_2_ (100 mM in HBS 1X pH 8.0) and of bovine serum albumin (BSA) (100 mg/mL) in HBS 1X pH 8.0 were made, sonicated and filtered through 0.2 µm filters. Stock solutions of BTB (5 mM) were dissolved in ethanol HBS 1X, and 10 mM HCl was diluted with deionized water. A liposomal POPC stock (0.1 mM) in HBS 1X pH 7.4 was made in a glass vial with 66.6 µL stock solution of POPC (3 mM) in chloroform: methanol (1/1 *v*/*v*) by evaporating; hydrating the dry lipid film with 2 mL HBS 1X stock; sonicated briefly for 1 min; free thawed 3 times in dry ice (acetone); and 40 °C water bath and extruding the hydrated film with an Avanti Mini-Extruder (610020) from Avanti Polar Lipids (Alabaster, AL, USA) through a 100 nm polycarbonate membrane. The size of POPC liposomes was determined with a Zeta sizer Nano (Malvern Panalytical, Westborough, MA, USA) by using the automated feature of the software and the volume reading option.

### 3.2. Methods

*Colorimetric enzyme activity assays*. *Hydrolysis of 4-nitrophenyl acetate (4NPA)* was followed spectrophotometrically by measuring the absorbance of released p-nitrophenyl acetate at 400 nm at 37 °C. In a typical assay, 3–5 µL enzyme stock (400 mU total activity) was diluted with buffer solution 1, pH 7.4 (except for CA, where a pH of 7.8 was used), to a final volume of HBS and a volume of 900 µL in a glass cuvette that was equilibrated for 10 min at 37 °C. A volume of 100 µL of 4-nitrophenyl acetate stock in DME (10 mM) was added to induce the enzymatic reaction (total volume of assay 1 mL and final concentration of 4-nitrophenyl acetate 1 mM), and absorbance was recorded every minute for 30 min of total reaction time. For the PLA2 assay, 5 µL of enzyme stock (80 U/mL) was mixed with 875 µL buffer solution 1, 10 µL CaCl_2_ 100 mM stock in HBS and 10 µL BSA stock in HBS. A parallel experiment without the enzyme was conducted to determine the rate of uncatalyzed hydrolysis reaction, which was deducted from the values for the catalyzed reaction for each enzyme. The amount of liberated 4-nitrophenol was determined from the absorbance values against a calibration curve.

*Hydrolysis of 4-nitrophenyl palmitate* was determined by the release of p-nitrophenol. Methods were adapted and modified [27,28,57,58]. Assay conditions were similar to the 4-nitrophenyl acetate assay measuring spectrophotometrically the absorbance at 400 nm at 37 °C. In a typical assay, 3–5 µL of enzyme stock (400 mU total activity) was diluted with buffer solution 1 pH 7.4 (except for CA, where pH of 7.8 was used) to a final volume of 900 µL in a glass cuvette that was equilibrated for 10 min at 37 °C. For the PLA2 assay, 5 µL of enzyme stock (80 U/mL) was mixed with 875 µL buffer solution 1, 10 µL CaCl_2_ 100 mM stock in HBS and 10 µL BSA stock in HBS. A volume of 100 µL of 4-nitrophenyl palmitate stock in 10% Triton X-100 (10 mM) was added to induce the enzymatic reaction, and the absorbance was recorded every minute for 30 min total reaction time. A parallel experiment without the enzyme was also conducted to determine the rate of uncatalyzed hydrolysis reaction, which was deducted from the values for the catalyzed reaction. The amount of 4-nitrophneol liberated was determined from the absorbance values against a calibration curve.

*Hydrolysis of 1,2-Di-O-lauryl-rac-glycero-3-(glutaric acid 6-methylresorufin ester) (DGGR).* The method was adapted and modified from several literature studies [39,42,44]. The hydrolysis of DGGR was determined spectrophotometrically at 37 °C, either via absorbance at 572 nm or via fluorescence (for example, 529 nm, Em. 600 nm, cutoff 590 nm). In a glass vial, 40 µL of DGGR stock in dioxane (2 mg/mL) was mixed with 955 µL of buffer 2 (pH = 8, except for CA, where pH 7.8 was used) solution under vigorous vortexing. For the PLA2 assay, 40 µL DGGR stock in dioxane (2 mg/mL) and 935 µL were mixed with 10 µL BSA stock and with 10 µL CaCl_2_ stock. The homogenous light orange solution was transferred to a quartz cuvette and equilibrated for 10 min at 37°. Enzymatic reactions were induced by adding 5 µL of enzyme stock, and the absorbance/fluorescence values were recorded every minute for 30 min total reaction time. A parallel experiment without the enzyme was conducted to determine the absorbance/fluorescence values of uncatalyzed reaction, which were deducted from the values for the catalyzed reactions for each enzyme. The amount of resorufin liberated was determined from the absorbance values against a calibration curve (Supplementary Materials, Figures S9 and S10).

*PLA2-specific assay*. In a typical assay, 465 µL of buffer 1 was mixed in a cuvette with 10 µL of BTB stock (5 mM), 10 µL of CaCl_2_ stock, 10 µL of BSA stock and 500 µL of 0.1 mM POPC liposome stock solution in HBS 1X. The homogenous green solution was equilibrated for 10 min at 37 °C, and the enzymatic reaction was induced by adding 5 µL of the PLA2 stock solution (80 U/mL). The hydrolysis reaction was followed spectrophotometrically by recording the absorbance at 427 nm and 620 nm every minute for a total of 30 min reaction time.

*Spectrum of BTB*. A spectrum of BTB was made by diluting 10 mM BTB stock solution 5 mM in ethanol to 0.01 mM (1000X) dilution using buffer 1 (HBS 1X pH 7.4). The diluted solution (1 mL) was transferred to a quartz cuvette, and the absorbance spectrum was recorded between 250 nm and 750 nm in 5 nm intervals at 37 °C.

*Calibration curve of BTB*. A calibration curve of BTB in HBS 1X pH 7.4 was produced by starting from a stock solution of 10 mM BTB in ethanol and using 5X, 10X, 20X, 100X, 150X, 200X, 500X and 1000X dilutions with HBS 1X pH 7.4. The absorbance of the diluted solutions of dye was recorded at 427 nm and 620 nm. In order to determine the linearity regime, a linear dependence of absorbance with concentrations of BTB was found in between 0.01 mM and 0.1 mM at 427 nm and 0.01 mM and 0.067 mM at 620 nm.

*Calibration of BTB response to acid produced*. In a quartz cuvette, 1 mL of BTB solution 0.05 mM in HBS 1 X pH 7.4 was prepared as described above, and it was treated with 10 µL aliquots of 10 mM HCl in DI water while recording the absorbance at 427 and 620 nm. The BTB response was found to be linear between 0 and 100 nmols of acid added.

*Calibration curve of BTB in the presence of acid*. A BTB calibration curve was produced to determine BTB absorbance variations in the presence of increased amounts of acid. A stock solution of 0.05 mM BTB in ethanol:HBS 1X was created by diluting 20X BTB stock with HBS 1x pH 7.4. In a quartz cuvette, 1 mL of 0.05 mM Bromothymol blue in HBS 1X was equilibrated for 10 min at 37 °C, and absorbance was recorded at 427 nm and 620 nm. Aliquots of 10 µL HCl 10 mM stock were added, and the absorbance was recorded at 427 nm and 620 nm after each addition. The absorbance at 427 nm and 620 nm was plotted (Figure 4A,B). Linearity was observed up to an amount of 1000 nmols of HCl added. R2 values were 0.9808 and 0.9877 (Figure 4A,B).

## 4. Conclusions

Various esterases present in the circulatory system have activities that are highly dependent on the lipophilicity of the substrate used to assess them. The activity observed for classical esterases was diminished when lipophilicity of the substrate increased, while for lipases, the activity observed generally increased following the interfacial activation model and depending highly on the type of lipase and its structure. The assays that we developed based on literature data allowed us to compare different esterases and lipases present in the circulatory system and to determine the most sensitive method for quantifying enzymatic activities against substrates of a particular type and lipophilicity.

## Data Availability

The data presented in this study are available in the article. Additional data are available from the corresponding author upon request.

## Supplementary Materials

The following supporting information can be downloaded at: https://www.mdpi.com/article/10.3390/ijms23031262/s1.
