# Supplementary material for: Evaluation of the Impact of Esterases and Lipases from the Circulatory System against Substrates of Different Lipophilicity
Source: Int J Mol Sci. 2022 Jan 23;23(3):1262. doi: 10.3390/ijms23031262 (PMC8836011; doi:10.3390/ijms23031262)
Supplement: Supplementary file 1 [file ijms-23-01262-s001.zip › ijms-1547573-supplementary.pdf]

Supplemental Material to

Evaluation of the impact of esterases from circulatory system against substrates of different lipophilicity

Leslie Lam[1] and Marc A. Ilies[2,3*]

[1]College of Science and Technology, Temple University, 1803 N. Broad Street Philadelphia, PA 19122.

[2]Department of Pharmaceutical Sciences and Moulder Center for Drug Discovery Research, Temple University School of Pharmacy, 3307 N Broad Street, Philadelphia, PA-19140.

[3]Lewis Katz School of Medicine, Alzheimer's Center (ACT), Temple University, Philadelphia, PA-19140, USA.

---

*Correspondence author: MAI: Phone: 215-707-1749; Fax 215-707-5620; Email: mailies@temple.edu;

Quantitation of 4-nitrophenol released during hydrolysis reactions

Calibration curves of 4-nitrophenol were done to determine the amount of 4-nitrophenol released from our enzymatic assays. Stock solutions of 10 mM 4-nitrophenol were made in HBS 1X pH 7.4 and pH 7.8 (in the case of CA) for quantitating the hydrolysis of 4-nitrophenyl acetate in our assay. Stock solutions pf 10 mM 4-nitrophenol was also made in 1% Triton X-100 HBS 1X pH 7.4 and 7.8 to quantitate the hydrolysis of 4-nitrophenyl palmitate. Stock solutions were vortexed and sonicated until all phenol dissolved. Serial dilutions (10X, 20X, 50X, 100X, 150X, 200X, 500X and 1000X) were performed on each stock solution of 4-nitrophenol with HBS 1X (pH 7.4 or pH 7.8 for CA). A volume of 1 mL of diluted sample was transferred to a quartz cuvette and incubated for 10 minutes at 37 ºC. The absorbance at 400 nm was read for all samples and plotted against corresponding concentration. The absorbance of buffer solution HBS 1X pH 7.4 and pH 7.8 were recorded at 400 nm at 37 ˚C and were deducted from each value obtained for the 4-nitrophenol solutions, and the corrected values were plotted to yield the calibration curves. Linearity was seen up to 0.20 mM for all 4 stock solutions of 4-nitrophenol. For calibration curves R^2 values were 0.9999, 0.9994, 0.9979, and 0.9999 (Figure S1, S2, S3, S4). The calibration curves were used to convert the absorbance of each enzymatic reaction in the hydrolysis of 4-nitrophenyl acetate and 4-nitrophenyl palmitate into amount of 4-nitrophenol (nmoles) released.

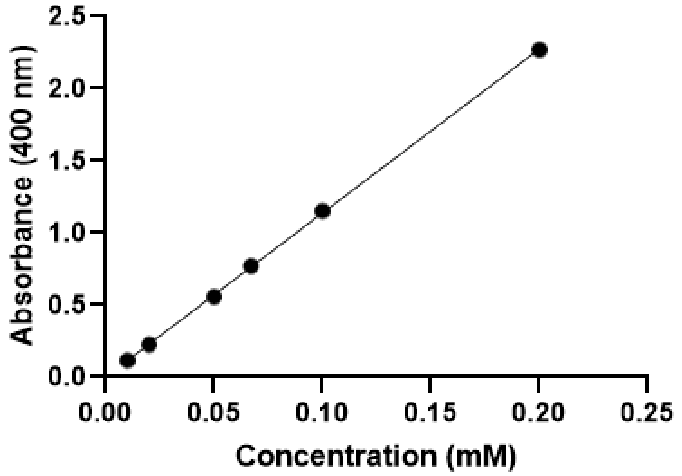

Figure S1: Calibration curve of 4-nitrophenol in HBS 1X pH 7.4, recorded at 400 nm and 37 ºC.

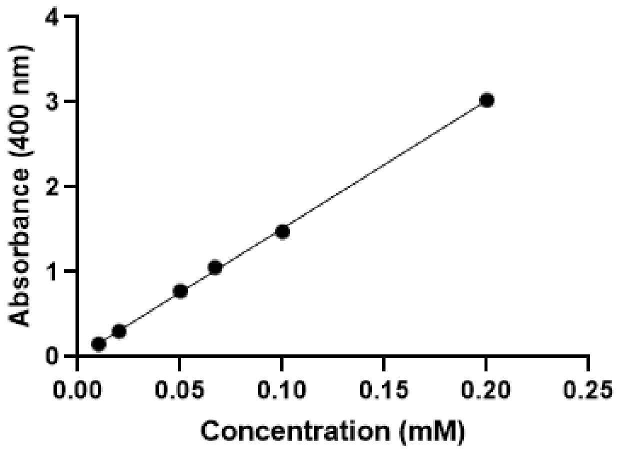

Figure S2: Calibration curve of 4-nitrophenol in HBS 1X pH 7.8, recorded at 400 nm and 37 ºC.

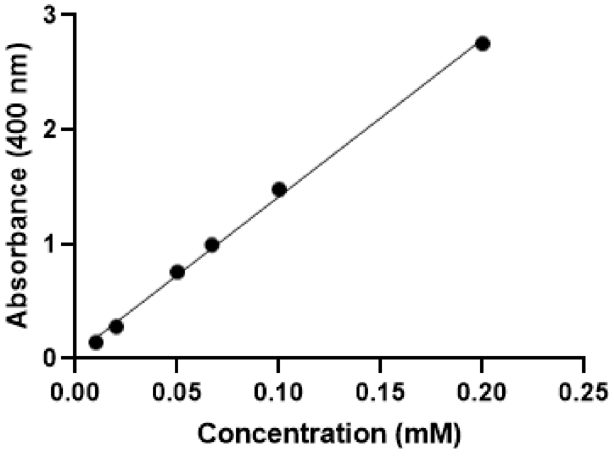

Figure S3: Calibration curve of 4-nitrophenol in 1% Triton X-100 HBS 1X pH 7.4, recorded at 400 nm and 37 ºC.

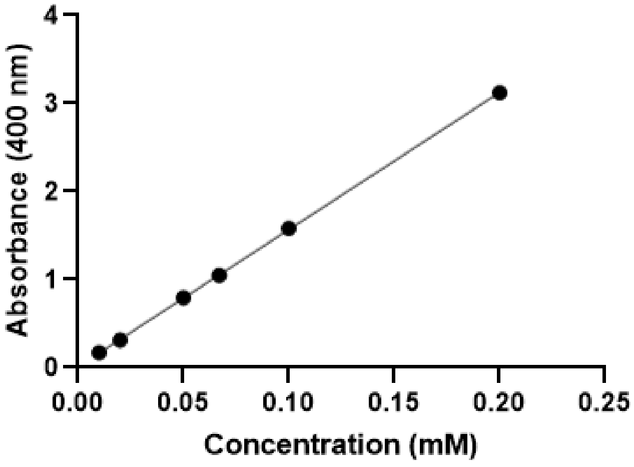

Figure S4: Calibration curve of 4-nitrophenol in 1% Triton X-100 HBS 1X pH 7.8, recorded at 400 nm at 37 ºC.

Spectrum of bromothymol blue (BTB). A spectrum of BTB was made by diluting 10 mM BTB stock solution 5 mM in ethanol to 0.01 mM final concentration (1000X dilution) using buffer 1 (HBS 1X pH 7.4). The diluted solution (1 mL) was transferred to a quartz cuvette and the absorbance spectrum was recorded between 250 nm-750 nm, in 5 nm intervals, at 37 °C (Figure S5).

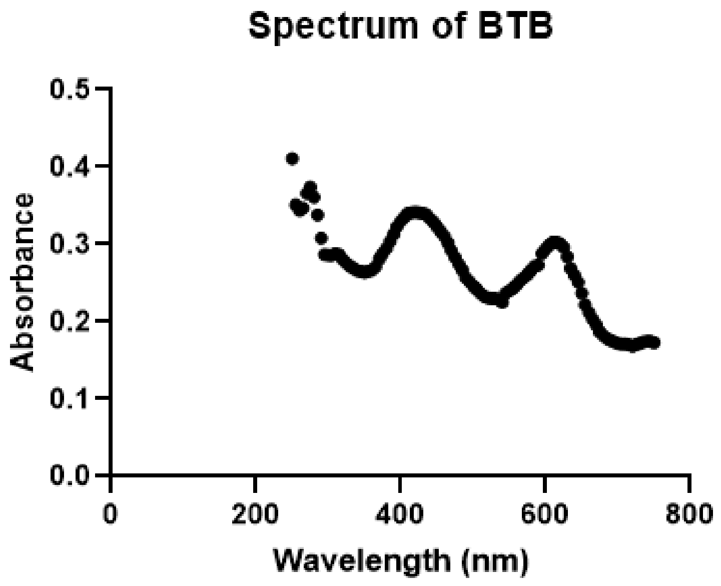

Figure S5: Spectrum of BTB stock (0.01 mM in HBS 1X) at 37 ºC.

Calibration curves for BTB were done in order to quantify the number of protons released in our PLA2 assay. A 10 mM stock solution was made by dissolving BTB in ethanol. Serial dilutions (5X, 10X, 15X, 20X, 50X, 100X, 150X, 200X, 500X and 1000X) were performed from the 10 mM BTB stock solution with HBS 1X pH 7.4. Diluted samples were transferred to a quartz cuvette, equilibrated for 10 minutes at 37 ˚C, and the absorbance was recorded at 427 nm and 620 nm. Absorbances were plotted against concentration (Figure S6 and S7). Calibration curves of BTB had R^2 values of 0.9935 and 0.9978. (Figure S6 and S7). Based on the linearity at 427 nm and 620 nm we decided to use 0.05 mM as our working concentration for BTB dye.

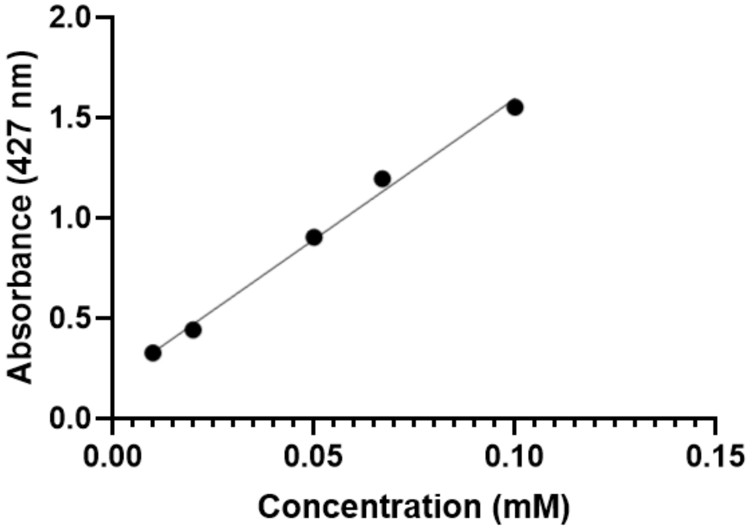

Figure S6: Calibration curve of BTB in ethanol:HBS 1X at 37 °C and 427 nm wavelength.

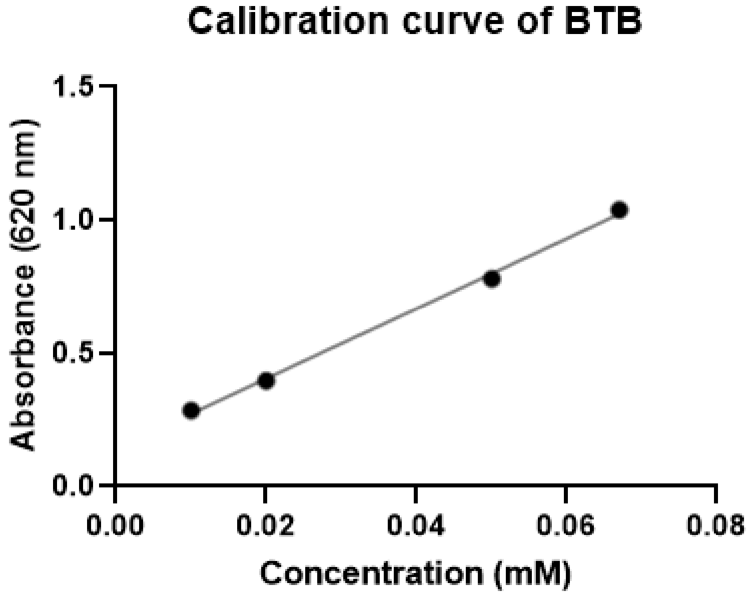

Figure S7: Calibration curve of BTB in ethanol:HBS 1X at 37 °C and 620 nm wavelength.

Calibration curve of BTB in presence of acid. BTB calibration curve was done to determine the BTB absorbance variation in the presence of increased amounts of acid. A stock solution of 0.05 mM BTB in ethanol:HBS 1X was made by diluting 20X BTB stock with HBS 1x pH 7.4 In a

quartz cuvette, 1 mL of 0.05 mM Bromothymol blue in HBS 1X was equilibrated for 10 minutes at 37 ºC, and the absorbance was recorded at 427 nm and 620 nm. Aliquots of 10 µL HCl 10 mM stock were added and the absorbance was recorded at 427 nm and 620 nm after each addition. The absorbance at 427 nm and 620 nm was plotted (Figures 4A and 4B). Linearity was seen up to an amount of 1000 nmols of HCl added. R^2 values were 0.9808 and 0.9877 (Figure 4 A&B).

BTB mediated HCl detection capacity. We decided to perform an experiment to determine if our working concentration of 0.05 mM BTB stock is enough to determine the total amount of acid generated in our enzymatic assay. For the assay, 465 µL of buffer 1 was mixed in a cuvette with 10 µL of BTB stock (5 mM), 10 µL of $CaCl_2$ stock, 10 µL of BSA stock, and 500 µL of 0.1 mM POPC liposome stock solution in HBS 1X. The homogenous green solution was equilibrated for 10 min at 37 °C and the enzymatic reaction was started by adding 5 µL of PLA2 stock solution (80 U/mL). The hydrolysis reaction was followed spectrophotometrically by recording the absorbance at 620 nm every minute, for 30 min total reaction time. After the assay was ran for 30 minutes, an amount of 20 µL of HCl 10 mM (200 nmols HCl) was added to the reaction and the absorbance was recorded for 10 minutes, reading it every minute. The absorbances was plotted against time (Figure S10). Based on data of Figure S10 the assay has excess capacity and can accommodate extra amounts of HCL, as seen, and therefore the enzymatic activity plateau is not due to the exhaustion of HCl titration capability of the assay.

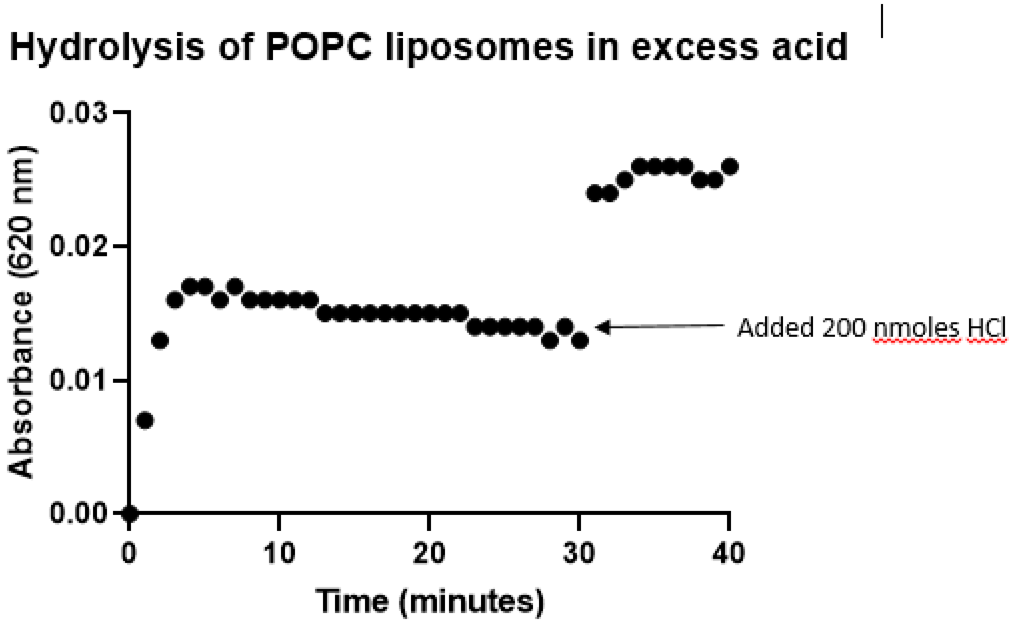

Figure S8: Activity of PLA2 from *Crotalus adamanteus* for hydrolysis of 0.1 mM POPC liposomes, 1 mM $CaCl_2$, 1 mg/mL BSA, and 0.05 mM BTB. Absorbance was recorded at 620 nm at 37 ºC. After 30 min reaction time, addition of 10 mM HCl was added to verify if there is excess capacity for HCL titration and absorbance was recorded for another 10 min.

<u>Calibration curves for resorufin</u> were done to determine the amount of resorufin released from our enzymatic assays. Stock solutions of 2 mg/mL resorufin were made in p-dioxane, and vortex and sonicated until dissolved. HBS 1X pH 7.4 and pH 7.8 (in the case of CA) for quantitating the hydrolysis of 4-nitrophenyl acetate in our assay. Serial dilutions (10X, 20X, 50X, 100X, 150X, 200X, 500X and 1000X, 1500X, 2000X, 10000X) were performed on each stock solution of resorufin with buffer 2 (pH 8.0 or pH 7.8 for CA). A volume of 1 mL of diluted sample was transferred to a quartz cuvette and incubated for 10 minutes at 37 ºC. The absorbance at 572 nm was read for all samples and plotted against corresponding concentration. The absorbance of buffer solution 2 pH 8.0 and pH 7.8 were recorded at 572 nm at 37 ˚C and were deducted

from each value obtained for the resorufin solutions, and the corrected values were plotted to yield the calibration curves. Linearity was seen up to 0.013 mg/mL for stock solutions pH 8.0 and pH 7.4. For calibration curves R^2 values were 0.9997 and 0.9958 (Figure S11, S12). The calibration curves were used to convert the absorbance of each enzymatic reaction in the hydrolysis of DGGR into amount of resorufin (nmols) released.

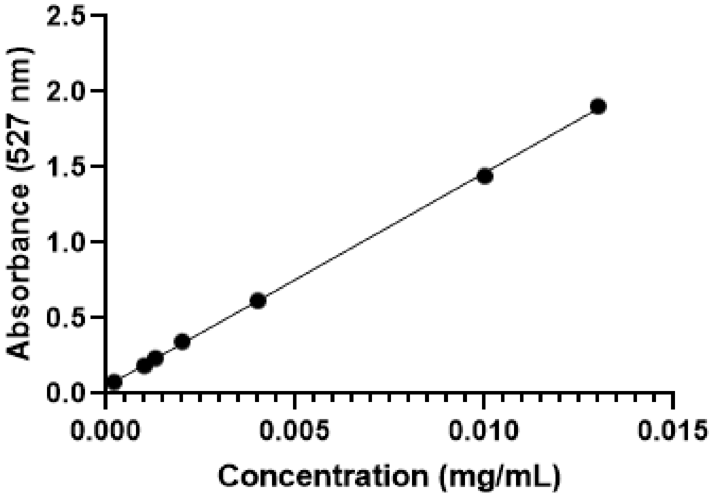

Figure S9: Calibration curve of resorufin in Buffer 2 pH 8.0, recorded at 37°C.

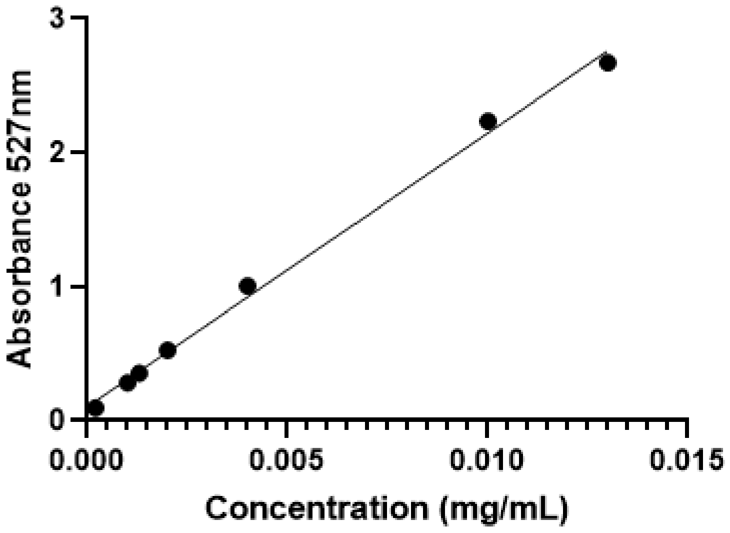

Figure S10: Calibration curve of resorufin in Buffer 2, pH 7.8, recorded at 37°C.
